# Association between food-based dietary inflammatory potential and ulcerative colitis: a case–control study

**DOI:** 10.1038/s41598-023-33138-7

**Published:** 2023-05-25

**Authors:** Zeinab Khademi, Parvane Saneei, Ammar Hassanzadeh-Keshteli, Hamed Daghaghzadeh, Hamid Tavakkoli, Peyman Adibi, Ahmad Esmaillzadeh

**Affiliations:** 1 Students’ Scientific Research Center, Tehran University of Medical Sciences, Tehran, Iran; 2 Department of Community Nutrition, School of Nutritional Sciences and Dietetics, Tehran University of Medical Sciences, Tehran, Iran; 3 Department of Community Nutrition, School of Nutrition and Food Science, Nutrition and Food Security Research Center, Isfahan University of Medical Sciences, Isfahan, Iran; 4 Department of Medicine, University of Alberta, Edmonton, Canada; 5 Integrative Functional Gastroenterology Research Center, Isfahan University of Medical Sciences, Isfahan, Iran; 6 Obesity and Eating Habits Research Center, Endocrinology and Metabolism Molecular -Cellular Sciences Institute, Tehran University of Medical Sciences, Tehran, Iran

**Keywords:** Gastrointestinal diseases, Nutrition

## Abstract

Despite several studies on the link between dietary inflammatory potential and risk of several conditions, limited studies investigated the association between pro-inflammatory diet and ulcerative colitis (UC). The objective of the present study was to examine the link between food-based dietary inflammatory potential (FDIP) and odds of UC in Iranian adults. This case–control study was carried out among 109 cases and 218 randomly chosen healthy controls. UC was diagnosed and confirmed by a gastroenterologist. Patients with this condition were recruited from Iranian IBD registry. Age- and sex-matched controls were selected randomly from participants of a large cross-sectional study. Dietary data were obtained using a validated 106-item semi-quantitative food frequency questionnaire (FFQ). We calculated FDIP score using subjects’ dietary intakes of 28 pre-defined food groups. In total 67% of subjects were female. There was no significant difference in mean age between cases and controls (39.5 vs. 41.5y; p = 0.12). The median (interquartile range) of FDIP scores for cases and controls were − 1.36(3.25) and − 1.54(3.15), respectively. We found no significant association between FDIP score and UC in the crude model (OR 0.93; 95% CIs 0.53–1.63). Adjustment for several potential confounders in multivariate model did not change this association (OR 1.12; 95% CIs 0.46–2.71). We failed to observe any significant association between greater adherence to a pro-inflammatory diet and risk of UC in this study. Prospective cohort studies are needed to further assess this relationship.

## Introduction

Ulcerative colitis (UC) is a chronic inflammatory condition of the gastrointestinal tract. In patients with UC, the superficial inflammation speared in an uninterrupted pattern starting from the rectum and may affect the entire colon^1,2^. Although inflammatory bowel disease (IBD) including UC was initiated in Western nations, nowadays newly industrialized countries in Asia and the Middle East have reported a rapid increase in its incidence^3^. In a national study, in Iran, it has been estimated that UC affects 35.52 per 100,000 subjects^4^.

The exact etiology of UC is yet to be determined, though, it is postulated that improper immune response, arising from a complex interaction between genetic susceptibility, an altered gut microbiota^5,6^, and several environmental factors^5^ might cause this condition. Dietary factors have been consistently found to modulate inflammation^7^, through which they might affect the development of chronic diseases^8,9^, including UC. Findings from previous studies indicated a positive link between adherence to a pro-inflammatory diet, assessed by dietary inflammatory index (DII), and odds of UC^10,11^. DII is a nutrient-based index constructed mostly based on pro-inflammatory or anti-inflammatory properties of nutrients^12^. It must be noted that people do not consume isolated nutrients rather they eat a diet consisting of numerous foods, nutrients, and other bioactive components with synergistic effects or interaction with each other^13,14^. Therefore, dietary inflammatory potential might be better reflected through a food-based index rather than a nutrient-based index. In 2016, Tabung et al.^15^ developed and validated an empirically dietary inflammatory pattern (EDIP) index using inflammatory biomarkers. Such assessment of dietary inflammatory potential has been examined in relation to ovarian and colorectal cancers^13,16^. Regarding IBD, in a recent prospective cohort, EDIP was significantly associated with risk of Crohn's disease (CD), but not UC^17^. Considering the paucity of information about the association between this dietary index and risk of UC in the Middle East, we aimed to investigate the association between dietary inflammatory potential, as assessed by a food-based dietary index, and risk of UC in a population-based case–control study in Iran.

## Methods

### Study participants

This population-based case–control study was performed among 109 patients with UC and 218 apparently healthy controls in Isfahan**,** Iran, between 2015 and 2019. The sample size was computed based on prior evidence indicating that approximately 60% of Iranian adults were following non-healthy dietary patterns^18^. According to earlier investigations in the country, we assumed that the consumption of unhealthy dietary patterns would double the risk of UC^10^. Therefore, with 80% study power and 5% type I error, and the ratio of controls to cases as 2, we reached the sample size of at least 101 cases and 202 controls for the current study. To enroll cases, we invited UC patients, whose information was registered in the IBD registry of Isfahan, to participate in a class intending to educate them about lifestyle modification. In that class, we mentioned the study and its aims and requested the participants to take part in the study. Out of all registered patients who attended that class (n = 140), 109 subjects agreed to take part in our study. All these patients provided written informed consent. To recruit controls, we used the study on the epidemiology of psychological, alimentary health, and nutrition (SEPAHAN) dataset. For each patient, two apparently healthy controls were randomly selected from SEPAHAN dataset on more than 8,000 people. Detailed information about the population-based cross-sectional study of SEPAHAN has been published elsewhere^19^. Controls were matched with cases in terms of age (± 2 years) and sex. Participants in the SEPAHAN study also provided informed written consent before enrollment^19^. Before random selection of controls from SEPAHAN dataset, we excluded individuals with any history of gastrointestinal disorders (including CD, UC, irritable bowel syndrome, functional dyspepsia, and gastroesophageal reflux disorder). The final sample size in the current study was 327 people (109 cases and 218 controls). This study was conducted according to the guidelines laid down in the Declaration of Helsinki and all procedures ethically approved by the Ethical Committee of the Tehran University of Medical Sciences, Tehran, Iran (IR.TUMS.VCR.REC.1398.497).

### Assessment of dietary intakes

Subjects were requested to complete a validated self-administrated 106-item dish-based semi-quantative food frequency questionnaire (DS-FFQ). This questionnaire was designed to be used in epidemiologic studies of Iranian population. Detailed data about this questionnaire has also been given elsewhere^20^. Foods and dishes included in that questionnaire were in five major categories: (1) 29 items of mixed dishes such as cooked or canned foods; (2) 10 items of grain-based foods and potatoes; (3) 9 items of dairy products; (4) 22 items of fruits and vegetables; and (5) 36 items of miscellaneous foods and beverages such as sweets, fast foods, nuts, desserts, and beverages. Nine multiple-choice frequency response categories, ranging from “never or less than once a month” to “12 or more times per day.” were available for each food item, allowing participants to report their usual daily intake. At last, data obtained from the FFQ was converted to grams per day for all participants via the booklet of household measures. Total daily energy and nutrient intakes were calculated using the modified US Department of Agriculture (USDA) food consumption database.

### Assessment of food-based dietary inflammatory potential

The construction of a food-based index to examine dietary inflammatory potential has been explained previously^21^. Briefly, data from a previous publication^22^ on 486 female teachers were used to identify foods, and food groups linked with serum C-reactive protein (CRP), interleukin-6 (IL-6), and tumor necrosis factor-α (TNF-α). Then using the factor loading values of foods and food groups provided by that publication^22^ and considering the quantity of consumption for each individual, the food-based dietary inflammatory potential (FDIP) index was constructed. In the present study at first, we computed mean daily intakes of pre-defined anti-inflammatory food groups (green leafy vegetables, cruciferous vegetables, yellow vegetables, tomatoes, other vegetables, fruits, fruit juices, poultry, fish, whole grains, legumes, and tea) and pro-inflammatory food groups (French fries, pizza, snacks, red meats, processed meats, eggs, refined grains, potatoes, dairy products, butter, hydrogenated oils, hydrogenated fats, mayonnaise, sweets and desserts, soft drinks, and coffee). And by applying the residual method^23^ energy-adjusted amounts of the above-mentioned food groups were calculated. Then, for each subject, we multiplied these amounts by their assigned factor loadings, obtained from that study^22^. Finally, we summed up the scores for each person to calculate total score of dietary inflammatory potential. To decrease the magnitude of the scores, the total scores were divided by 100 for all participants.

### Assessment of UC

UC was diagnosed by an experienced gastroenterologist according to physical, colonoscopic, and histological examinations. We additionally reviewed medical records to confirm the diagnosis.

### Assessment of other variables

Data on other variables including age, sex, education, smoking status, and diabetes history were collected through a pre-tested structured self-administered questionnaire. We used a pretested dietary habit questionnaire to obtain information regarding dietary habits including meal regularity, fluid consumption during meals, chewing efficiency, fried foods intake, and fatty meals intake. To assess physical activity, we applied General Practice Physical Activity Questionnaire (GPPAQ)^24^, and then classified study subjects into five categories: no activity, < 3 h per week, 3–5 h per week, 5–7 h per week, and $\ge$ 7 h per week. Other required data including weight and height were collected using a self-administered questionnaire. In a recent study from our group, it was found that anthropometric data driven from self-reported questionnaires provided valid information compared with actual measured values^25^. Body mass index (BMI) was calculated as weight (kg) divided by height square (m^2^).

### Statistical analysis

The normal distribution of the data was evaluated using the Kolmogorov– Smirnov normality test. We classified all study participants according to defined tertile cut-off points for FDIP score in the control group. The distribution of cases and controls by continuous and categorical variables were assessed using student’s t-test and chi-square test, respectively. To examine the differences across tertiles of FDIP score, we applied analysis of variance (ANOVA) for continuous variables and chi-square test for categorical variables. Analysis of covariance (ANCOVA) was used to compare energy, age, and sex-adjusted dietary intakes of subjects across tertiles of FDIP score. Association between adherence to a pro-inflammatory diet and UC was examined in multivariable-adjusted models, using binary logistic regression. We adjusted for age (continuous) and sex (female/male) in the first model and additionally for total energy intake (calory/day) and BMI (continuous) in the second model. Further adjustment for education (high school diploma or below/university graduated), smoking status (non-smoker/smoker), diabetes history (yes/no), and physical activity (no activity/ < 3 h per week/3–5 h per week/5–7 h per week/$\ge$ 7 h per week) was done in the third model. We also adjusted for regular meal consumption (often or always/never or occasionally), fluid consumption during meals (< 3 glasses/ ≥ 3 glasses), chewing efficiency (a lot/not a lot), fried foods intake (< 4 per week/ ≥ 4 per week), and consumption of fatty meals (non-fatty meal/fatty meal) in the last model. The first tertile of FDIP score was considered the reference category, in all analyses. By treating tertile of FDIP score as an ordinal variable, P for trends was determined. All statistical analyses were carried out using SPSS software version 19. P values were considered significant at < 0.05.

## Results

Subjects with UC were less likely to be physically active (17% vs. 32%; *P* = 0.02) and university graduates (38% vs. 54%; *P* = 0.007) than controls. No significant differences in mean age and BMI were seen between cases and controls. We failed to find any significant differences between cases and controls, comparing them in terms of sex, smoking status, and history of diabetes. Comparing cases and controls in terms of dietary habits of chewing efficiency, regular meal pattern, fried foods intake, fluid consumption during meals, and fatty meals intake, no significant differences were found.

UC cases reported higher intakes of total energy (3014 ± 101 vs. 2328 ± 69; *P* < 0.001), polyunsaturated fatty acid (PUFA) (43 ± 1.08 vs. 31 ± 0.72; *P* < 0.001), yellow vegetables (16.03 ± 1.01 vs. 6.54 ± 0.68; *P* < 0.001), cruciferous vegetables (8.97 ± 0.91 vs. 3.89 ± 0.61; *P* < 0.001), vegetable oils (52.0 ± 1.71 vs. 42.8 ± 1.16; *P* < 0.001), and red meat (85.6 ± 4.15 vs. 73.9 ± 2.80; *P* = 0.02) as well as lower intakes of protein (89 ± 1.80 vs. 95 ± 1.21; *P* < 0.001), dietary fiber (18 ± 0.67 vs. 24 ± 0.45; *P* < 0.001), monounsaturated fatty acid (MUFA) (29 ± 1.02 vs. 40 ± 0.68; *P* < 0.001), other vegetables (128.1 ± 7.13 vs. 146.9 ± 4.64; *P* = 0.03), hydrogenated fats (0.35 ± 0.44 vs. 5.34 ± 0.30; *P* < 0.001), and refined grain (286.4 ± 17.4 vs. 346.3 ± 11.6; *P* = 0.005) than controls.

Participants in the top tertile of FDIP score were more likely to be younger and had lower BMI. No other significant differences were observed in terms of other main characteristics across tertiles of FDIP score. Comparing study participants across tertiles of FDIP score, we found no significant associations regarding dietary habits (Table 1).

**Table 1:** General characteristics and dietary habits of study participants across tertiles of FDIP score.

	Tertiles of FDIP score			*P*^†^
	T1	T2	T3	
Subjects, n	112	107	108	
Age, y	42.4 ± 10.4	40.8 ± 10.6	37.1 ± 10.6	0.001
BMI,$\mathrm{kg}/{\mathrm{m}}^{2}$	25.3 ± 3.5	25.9 ± 4.0	24.5 ± 3.5	0.04
Sex (Females), %	60	51	54	0.09
Smoking status (current smoker), %	3	6	5	0.68
History of diabetes (yes), %	3	3	3	0.99
Education (University graduate), %	51	46	49	0.74
Physical activity, %				0.04
No activity	7	3	12	
$\le$ 3 h per week	28	27	37	
3–5 h per week	19	33	19	
5–7 h per week	15	6	13	
$\ge$ 7 h per week	31	31	19	
Regular meal pattern (Often or always), %	63	65	65	0.97
Chewing sufficiency (A lot), %	17	8	19	0.71
Fluid consumption $(\ge$ 3 glasses), %	3	5	4	0.70
Consumption of fried foods ($\ge$ 4 per week), %	15	10	21	0.20
Consumption of fatty meals (Fatty meal), %	2	3	1	0.59

*FDIP* food-based dietary inflammatory potential.

Data are presented as mean ± standard deviation (SD) or percent.

^†^Obtained from ANOVA or chi-square test, where appropriate.

Subjects with greater adherence to a pro-inflammatory diet (tertile 3), had higher intakes of total energy, carbohydrates, and refined grains and lower intakes of total fats, PUFA, MUFA, fruits, fruit juices, legumes, cruciferous vegetables, green leafy vegetables, yellow vegetables, other vegetables, tea, tomatoes, and whole grains than those with the least adherence (Table 2).

**Table 2:** Dietary intakes of study participants across tertiles of FDIP score.

	Tertiles of FDIP score			*P*^†^
	T1	T2	T3	
Energy (Kcal/d)	2560 ± 94	2210 ± 94	2655 ± 97	0.003
Carbohydrates (g/d)	308 ± 5.92	284 ± 6.04	311 ± 6.14	0.003
Protein (g/d)	91.7 ± 1.64	93.3 ± 1.67	90.3 ± 1.70	0.47
Total Fats (g/d)	102 ± 2.27	110 ± 2.31	98.4 ± 2.35	0.001
SFA (g/d)	24.03 ± 0.55	25.29 ± 0.57	23.95 ± 0.58	0.17
PUFA (g/d)	33.97 ± 1.17	37.36 ± 1.19	32.28 ± 1.21	0.01
MUFA (g/d)	35.58 ± 1.003	39.06 ± 1.02	34.44 ± 1.04	0.005
Total fiber (g/d)	24.43 ± 0.55	20.84 ± 0.56	17.53 ± 0.57	< 0.001
Fruit (g/d)	415.9 ± 14.9	219.7 ± 15.3	142.6 ± 15.6	< 0.001
Fruit juice (g/d)	54.3 ± 5.74	38.3 ± 5.84	28.4 ± 6.0	0.008
Poultry (g/d)	57.1 ± 3.99	55.1 ± 4.07	51.2 ± 4.14	0.58
Legumes (g/d)	42.5 ± 3.50	53.4 ± 3.57	40.4 ± 3.64	0.04
Cruciferous vegetable (g/d)	8.59 ± 0.86	5.40 ± 0.88	2.29 ± 0.90	< 0.001
Green leafy vegetable (g/d)	23.3 ± 1.97	14.6 ± 2.01	7.05 ± 2.06	< 0.001
Yellow vegetable (g/d)	12.8 ± 1.02	9.58 ± 1.04	6.10 ± 1.05	< 0.001
Other vegetables (g/d)	174.5 ± 6.16	138.1 ± 6.31	109.6 ± 39	< 0.001
Tea (g/d)	467.4 ± 25.1	310.6 ± 25.5	259.4 ± 26.1	< 0.001
Tomatoes (g/d)	85.1 ± 7.32	63.3 ± 4.44	47.4 ± 4.55	< 0.001
Whole grains (g/d)	62.1 ± 7.32	43.1 ± 7.50	27.7 ± 7.65	0.006
Butter (g/d)	0.70 ± 0.16	0.83 ± 0.17	1.23 ± 0.89	0.08
Potatoes (g/d)	54.1 ± 3.28	60.5 ± 3.35	51.8 ± 3.41	0.17
Diary (g/d)	211.7 ± 17.7	195.5 ± 18.1	244.6 ± 18.3	0.16
Fish (g/d)	20.0 ± 4.95	19.5 ± 5.05	15.0 ± 5.14	0.75
Refined grains (g/d)	252.3 ± 15.3	293.9 ± 15.1	434.3 ± 15.2	< 0.001
Red meats (g/d)	71.8 ± 3.97	85.3 ± 4.05	76.2 ± 4.11	0.05
Processed meats (g/d)	5.11 ± 0.68	6.83 ± 0.69	5.86 ± 0.7	0.21
Sweets & desserts (g/d)	15.5 ± 2.31	17.1 ± 2.37	21.1 ± 2.42	0.24
Pizza (g/d)	10.7 ± 1.27	14.4 ± 1.30	11.7 ± 1.33	0.11
Eggs (g/d)	26.8 ± 2.12	26.2 ± 2.17	25.9 ± 2.20	0.94
Soft drinks (g/d)	14.6 ± 4.36	19 ± 4.44	27.6 ± 4.55	0.11
Snacks (g/d)	1.95 ± 0.30	1.59 ± 0.31	1.16 ± 0.31	0.20
French fries (g/d)	7.31 ± 1.08	9.01 ± 1.11	6.70 ± 1.12	0.32
Coffee (g/d)	8.72 ± 2.35	8.89 ± 2.40	6.71 ± 2.46	0.78
Mayonnaise (g/d)	3.85 ± 0.96	4.62 ± 0.98	4.07 ± 0.49	0.99
Hydrogenated fats (g/d)	3.74 ± 0.47	3.82 ± 0.49	3.74 ± 0.49	0.99
Vegetable oils (g/d)	43.4 ± 1.68	48.8 ± 1.71	44.9 ± 1.74	0.07

*FDIP* food-based dietary inflammatory potential, *SFA* saturated fatty acid, *PUFA* polyunsaturated fatty acid, *MUFA* monounsaturated fatty acid.

^†^All values were adjusted for age, sex, and energy, except for dietary energy intake, which was only adjusted for age and sex using ANCOVA.

Neither in crude nor in multivariable-adjusted models, we found a significant association between FDIP score and UC. In model 1, after adjustment for sex and age, no significant association between FDIP score and UC was observed (OR 1.004; 95% CI 0.55–1.82). Further adjustments for total energy intake and BMI in model 2 (OR 0.98; 95% CI 0.51–1.89), and additionally controlling for educational status, smoking status, diabetes history, physical activity, and total fiber intake in model 3 (OR 1.07; 95% CI 0.46–2.47) did not change the association. In the last model, we also took dietary habits into account, but no significant link was found (OR 1.12; 95% CI 0.46–2.71) (Table 3).

**Table 3:** Multivariable-adjusted ORs and 95% CIs for UC across tertiles of FDIP score.

	Tertiles of FDIP score			P-trend
	T1	T2	T3	
Ulcerative colitis				
Crude	1.00	0.87 (0.49–1.53)	0.93 (0.53–1.63)	0.81
Model 1^a^	1.00	0.91 (0.51–1.63)	1.004 (0.55–1.82)	0.99
Model 2^b^	1.00	1.13 (0.60–2.11)	0.98 (0.51–1.89)	0.99
Model 3^c^	1.00	0.95 (0.40–2.27)	1.07 (0.46–2.47)	0.87
Model 4^d^	1.00	0.88 (0.35–2.19)	1.12 (0.46–2.71)	0.81

*FDIP* food-based dietary inflammatory potential.

Data are OR (95% CI).

^a^Model 1: Adjusted for age, and sex.

^b^Model 2: Further, adjusted for total energy intake, and BMI.

^c^Model 3: Further, adjusted for education, smoking, diabetes history, physical activity, and total fiber intake.

^d^Model 4: Further, adjusted for regular meal pattern, chewing sufficiency, fluid consumption during a meal, fried food intake, and fatty food intake.

## Discussion

In the present study, we failed to find a significant association between FDIP score and odds of UC. Adjustment for a wide range of potential covariates did not change the association. This study is among the first studies investigating FDIP score in relation to UC risk.

Traditionally known as a Western disease, UC incidence is increasing worldwide, causing a major public health burden and reducing quality of life for those who suffer from this condition^5^. Despite growing investigations examining dietary patterns in relation to UC risk, findings are still conflicting. In 2014, Racine et al.^26^ found a link between the “high sugar and soft drinks” pattern and an increased risk of UC incidence only in subjects with low vegetable intake in the EPIC cohort. In another cohort study examining adherence to the Mediterranean dietary pattern and later onset of UC, no significant association was observed^27^. Findings of the NutriNet-Sante cohort also revealed no link between 3 dominant retained dietary patterns:” healthy”, “traditional”, and “western” with UC incidence^28^.

In this study, we found no significant association between adherence to a pro-inflammatory dietary pattern and UC risk. In agreement with our findings, in a recent analysis of 3 prospective cohort studies, dietary patterns with high inflammatory potential were not significantly linked with UC development^17^. On the contrary, our previous analysis of this group of individuals revealed a positive link between nutrient-based dietary inflammatory potential and risk of UC^11^. Shivappa et al.^10^ also observed a positive association between higher DII score and odds of UC in the framework of a hospital-based case–control study in Iran. The reason behind these conflicting findings might be due to different approaches used to assess the inflammatory potential of the diet in different studies. In this study, unlike previous ones^10,11^, we used a food-based dietary index to examine inflammatory potential of diet because nutrient-based DII cannot capture complex interactions within the whole diet^29–31^. In the past, undernutrition and nutritional deficiencies were among leading diet-induced diseases, this shifted epidemiological studies to focus on nutrients and their contribution to health outcomes. Nowadays, this approach failed considering the rising incidence of chronic diseases^32,33^, therefore focusing only on nutrient-disease links might lead to finding biased associations. Additional studies in this field are needed to reach definite findings.

Though we did not observe any significant findings, there are plausible mechanisms explaining how adherence to a pro-inflammatory diet might contribute to the etiology of IBD. For instance, it is known that cytokines are directly involved in the development, expansion, and maintenance of UC^34–36^. Therefore, a pro-inflammatory diet resulting in increased cytokine production might be involved in UC pathogenesis. Considering the critical role of gut microbiota in the healthy intestine, a diet rich in food items with inflammatory properties may promote intestinal inflammation by altering gut microbiota composition^37–39^.

Lack of finding any association in this study might be attributed to the nature of IBD. Interestingly, in several previous publications, investigators found diet and its components to be associated with CD risk and not UC^40,41^. It seems that diet is a more significant risk factor for CD than UC^42^, due to the more significant role of gut microbiome in CD pathogenesis and the ability of dietary components to modulate the composition of gut microbiota^43^. Lack of finding any association in regression models might also be due to lack of power, given the low number of study participants.

Our study has several strengths as well as limitations. Being among the first studies in this field, applying a validated FFQ for dietary assessment, and adjustment for several potential covariates are among the strengths. Moreover, in this study, we used a food-based index to determine the inflammatory potential of the diet, which can capture complex interactions within the whole diet. However, some limitations should be also noted. First, due to the case–control design of this study, we were not able to establish casualty. Second, recall and selection biases are common in case–control studies and might affect our findings. Third, despite adjustment for several potential covariates, the effect of residual confounders such as history of medication (e.g., non-steroidal anti-inflammatory drugs (NSAIDs)) intake cannot be ignored. Forth is the small sample size of the present study. Finally, the possibility of diet alteration in UC patients due to their condition should also be kept in mind.

In conclusion, we failed to observe any significant link between adherence to a pro-inflammatory diet, measured by FDIP, and odds of UC. Additional adjustments for several potential covariates did not change the result. Prospective cohort studies are needed to confirm these findings.

## Data Availability

The datasets used and analyzed during the current study are available from the corresponding author upon reasonable request.

## Abbreviations

UC: Ulcerative colitis
IBD: Inflammatory bowel disease
DII: Dietary inflammatory index
EDIP: Empirically dietary inflammatory pattern
CD: Crohn's disease
SEPAHAN: Study on the epidemiology of psychological, alimentary health, and nutrition
DS-FFQ: Dish-based semi-quantitative food frequency questionnaire
USDA: US department of agriculture
CRP: C-reactive protein
IL-6: Interleukin-6
TNF-α: Tumor necrosis factor-α
FDIP: Food-based dietary inflammatory potential
GPPAQ: General practice physical activity questionnaire
BMI: Body mass index
ANOVA: One-way analysis of variance
ANCOVA: Analysis of covariance
OR: Odds ratio
CI: Confidence interval
NSAIDs: Non-steroidal anti-inflammatory drugs
